# Comparison of three rapid diagnostic tests for bloodstream infections using Benefit-risk Evaluation Framework (BED-FRAME)

**DOI:** 10.1128/jcm.01096-23

**Published:** 2023-12-06

**Authors:** Richard D. Smith, Min Zhan, Shanshan Zhang, Surbhi Leekha, Anthony Harris, Yohei Doi, Scott Evans, J. Kristie Johnson, Robert K. Ernst

**Affiliations:** 1 Department of Pathology, University of Maryland School of Medicine, Baltimore, Maryland, USA; 2 Department of Microbial Pathogenesis, University of Maryland School of Dentistry, Baltimore, Maryland, USA; 3 Department of Epidemiology and Public Health, University of Maryland School of Medicine, Baltimore, Maryland, USA; 4 Biostatistics Center and the Department of Biostatistics and Bioinformatics, The George Washington University, Washington, D.C., USA; 5 Division of Infectious Diseases, University of Pittsburgh School of Medicine, Pittsburgh, Pennsylvania, USA; Maine Medical Center Department of Medicine, Portland, Maine, USA

**Keywords:** rapid diagnostic testing, bloodstream infections, blood culture, BED-FRAME

## Abstract

Rapid diagnostic tests (RDTs) for bloodstream infections have the potential to reduce time to appropriate antimicrobial therapy and improve patient outcomes. Previously, an in-house, lipid-based, matrix-assisted laser desorption/ionization-time of flight mass spectrometry (MALDI-TOF MS) method, Fast Lipid Analysis Technique (FLAT MS), has shown promise as a rapid pathogen identification method. In this study, FLAT MS for direct from blood culture identification was evaluated and compared to FDA-cleared identification methods using the Benefit-risk Evaluation Framework (BED-FRAME) analysis. FLAT MS was evaluated and compared to Bruker Sepsityper and bioMérieux BioFire FilmArray BCID2 using results from a previous study. For this study, 301 positive blood cultures were collected from the University of Maryland Medical Center. The RDTs were compared by their sensitivities, time-to-results, hands-on time, and BED-FRAME analysis. The overall sensitivity of all platforms compared to culture results from monomicrobial-positive blood cultures was 88.3%. However, the three RDTs differed in their accuracy for identifying Gram-positive bacteria, Gram-negative bacteria, and yeast. Time-to-results for FLAT MS, Sepsityper, and BioFire BCID2 were all approximately one hour. Hands-on times for FLAT MS, Sepsityper, and BioFire BCID2 were 10 (±1.3), 40 (±2.8), and 5 (±0.25) minutes, respectively. BED-FRAME demonstrated that each RDT had utility at different pathogen prevalence and relative importance. BED-FRAME is a useful tool that can used to determine which RDT is best for a healthcare center.

## INTRODUCTION

Rapid diagnostic tests (RDTs) can optimize the management of bloodstream infections (BSIs) by reducing the time to appropriate antimicrobial treatment (1–3). RDTs are associated with improved patient outcomes, including decreased mortality, morbidity, and hospitalization (4). Because of these benefits, RDTs have become prominent in clinical microbiology laboratories. Currently, several RDTs for identifying pathogens causing BSIs exist with more in development (5).

One technology in development is lipid-based, matrix-assisted laser desorption/ionization-time of flight mass spectrometry (MALDI-TOF MS) (6). Previously, our group had developed a lipid-based extraction method, Fast Lipid Analysis Technique (FLAT), which when paired with lipid-based MALDI-TOF MS (FLAT MS) allows for pathogen identification using microbial membrane lipids. FLAT MS has demonstrated the ability to accurately identify pathogens directly from biological specimens, such as urine, and limited resistance markers within one hour (7–9). However, the accuracy of FLAT MS for blood cultures in a clinical setting has not been previously studied. In this study, FLAT MS was used to identify pathogens directly from positive blood culture (PBC).

With the emergence of novel RDTs for BSIs, such as FLAT MS, and their potential impact on patient outcomes, it is imperative to compare novel and existing RDTs. Traditional statistics for evaluating and comparing different diagnostic methods include sensitivity, specificity, positive and negative predictive values, likelihood ratios, time-to-results, and overall accuracy (10). Despite the insight these statistics provide, choosing the best diagnostic test for a particular healthcare setting remains difficult as these evaluation methods fail to incorporate local epidemiology and provider preferences. To address these limitations, the Antibiotic Resistance Leadership Group has developed the Benefit-risk Evaluation for Diagnostics Framework (BED-FRAME) (11). BED-FRAME supplements traditional evaluation methods by illustrating and estimating diagnostic yield based on local prevalence and perceived relative importance of potential errors to clinicians, thus helping understand the clinical impact of each diagnostic. However, there are no published studies comparing diagnostics using BED-FRAME with real data.

We conducted a proof-of-concept study of the comparison of RDTs incorporating methods beyond traditional evaluation. There were two objectives of this study. First, evaluate the performance of FLAT MS for identifying pathogens directly from PBC and compare its performance to FDA-cleared RDTs for identification directly from PBC {Smith, 2023 #235}. Second, use BED-FRAME methodology to compare these RDTs and determine which RDT to implement at an academic medical center.

## MATERIALS AND METHODS

### Population and study setting

We included 301 PBCs, including polymicrobial cultures from adult patients (age ≥18 years) at the University of Maryland Medical Center (UMMC) from September 2021 to August 2022 (IRB HP-76072), with only the first PBC from a patient included. PBCs were processed using each of the three assays within 12 hours of positivity {Smith, 2023 #235}. Collection and processing of PBCs was conducted by research team members and was limited to Monday through Friday from 8 a.m. to 5 p.m.

### FLAT extraction and lipid-based MALDI-TOF MS analysis

**Fig 1:**
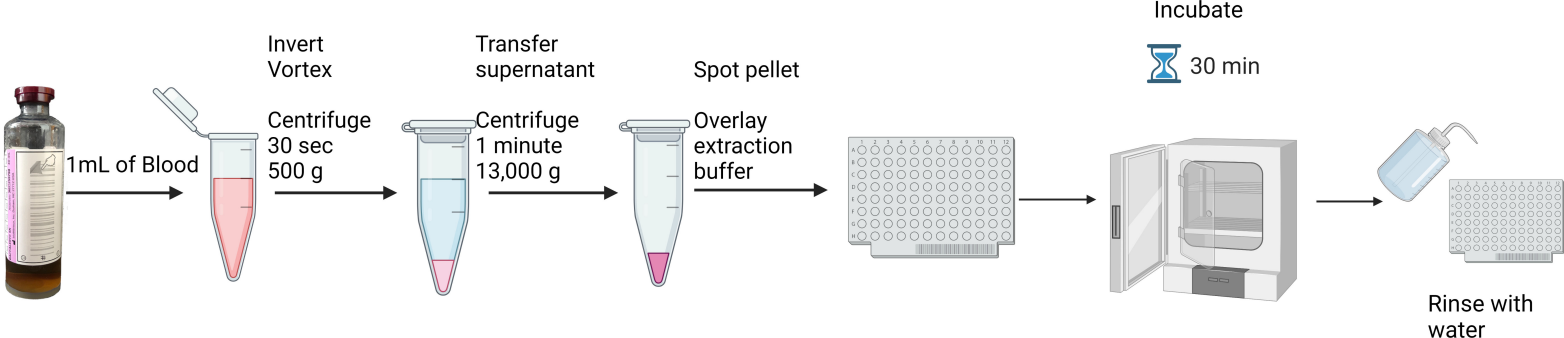
FLAT extraction protocol for direct from positive blood culture analysis using lipid-based MALDI-TOF MS. Extraction allows for results in under an hour and hands-on time less than 10 minutes.

For FLAT extraction, 1 mL of PBCs was transferred to a 1.5-mL tube and centrifuged for 30 seconds at 500 × g to remove red blood cells. The supernatant was transferred to a new tube, centrifuged for 1 minute at 13,000 × g, and the supernatant was discarded. The pellet was smeared in two wells on a MALDI target plate, 1 µL of 10 mM, pH 4.5 citric acid extraction buffer made in-house was placed on top of the sample, and the target plate was incubated in a laboratory incubator at 110°C for 30 minutes and rinsed with 500 µL of endotoxin-free water (Fig. 1) followed by 1  µL of 10 mg/mL norharmane matrix for each sample. Analysis was conducted using the FDA-approved MALDI Biotyper Sirius system (MBT) (Bruker Daltonics, Billerica, MA) in negative-ion linear mode. Spectra were collected between *m/z* 1,000 and *m/z* 2,400 (6).

### Bruker sepsityper

The Sepsityper kit (Bruker Daltonics) consists of two extraction methods: Rapid Sepsityper and Sepsityper. Both extraction methods were performed as previously published (12). Samples were analyzed using the FDA-approved MBT.

### BioFire FilmArray BCID_2_

The BioFire BCID2 (bioMérieux Inc., Durham, NC) was run on the first PBC per patient as per routine at the UMMC clinical microbiology laboratory {Smith, 2023 #235}. Once positive, 4 to 5 drops of the PBC were mixed with the sample buffer along with a hydration solution and inserted into the pouch. The reagent pouch was then inserted into the instrument (13).

### Traditional evaluation of diagnostic tests

Traditional evaluation methods included sensitivity, time-to-results, and hands-on time. Specificity was not calculated as we only evaluated PBC samples. Furthermore, while false positives can occasionally occur when organisms are identified that are not recovered in culture, this was not observed in this study, making the calculation of specificity challenging. Identification from each test was compared to standard culture-based identification at the genus and species levels. If enteric pathogens were identified as “Enterobacterales” using BioFire BCID2, it was not considered an accurate identification. For monomicrobial cultures, sensitivities were stratified by Gram-positive bacteria, Gram-negative bacteria, and yeast. For polymicrobial cultures, the RDTs were compared by the proportion of complete identification, all organisms present in culture were identified, or partial identification, at least one but not all organisms were identified. Time-to-results was the time from the beginning of the procedure to when the results were provided. Hands-on time was the time required for processing by the laboratory staff. Results for Sepsityper and BioFire BCID2 were taken from a previous study conducted by our group {Smith, 2023 #235}. FLAT MS was performed in parallel with Sepsityper and BioFire BCID2.

### BED-FRAME

BED-FRAME consists of five steps that convey potential differences in clinical impact among different diagnostics (11). The original framework of this analysis was established for choosing between two diagnostics, where one had higher sensitivity and the other had higher specificity. However, the goal of this analysis was to compare the clinical impact of these RDTs based on their differing abilities to accurately identify monomicrobial Gram-positive and Gram-negative bacteria. For this analysis, yeast was excluded due to sample size limitations. Polymicrobial samples, for which clinical laboratories may not prioritize polymicrobial PBCs as they are rare and frequently considered contamination, were also excluded from the analysis (14). Because the three RDTs compared in this study have equivalent overall accuracies for monomicrobial PBCs and similar time-to-results, deciding which test to implement may remain difficult for laboratories that prioritize monomicrobial cultures. Therefore, the original BED-FRAME equations were modified to address the study’s goal.

#### Step 1: Displaying expected yield as a function of prevalence

Diagnostic yield is the proportion of true positives, true negatives, false positives, and false negatives. For this study, these values were “accurately identified Gram-positive bacteria,” “accurately identified Gram-negative bacteria,” “unidentified Gram-positive bacteria,” and “unidentified Gram-negative bacteria” and were calculated as follows:

Accurately identified Gram-positive bacteria = (Gram-positive sensitivity) × (Prevalence Gram-positive) × (population size)

Accurately identified Gram-negative bacteria = (Gram-negative sensitivity) × (1 − Prevalence Gram-positive) × (population size)

Unidentified Gram-positive bacteria = (1 − Gram-positive sensitivity) × (Prevalence Gram-positive) × (population size)

Unidentified Gram-negative bacteria = (1 − Gram-negative sensitivity) × (1 − Prevalence Gram-positive) × (population size)

Gram-positive bacterial prevalence ranged from 0% to 100% where 0% represents a population of approximately 100% Gram-negative bacteria. The population was fixed to 1000 PBCs.

#### Step 2: Plot the expected diagnostic difference in unidentified Gram-negative bacteria and accurately identify Gram-positive bacteria as a function of prevalence

Using the expected diagnostic yield calculated in step 1, the expected difference in raw numbers of unidentified Gram-negative bacteria and accurately identified Gram-positive bacteria between two tests was plotted as a function of prevalence for each possible comparison: Sepsityper and BioFire, Sepsityper and FLAT, and BioFire and FLAT.

#### Step 3: Calculate the number needed to test (NNT)

NNT is the number of patients who are positive for a disease that must be tested with one test relative to another test, to result in one additional true positive result. NNT was calculated for Gram-positive (NNT_GP_) and Gram-negative bacteria (NNT_GN_). NNT_GP_ was the number of PBCs containing Gram-positive bacteria that must be tested with one test versus another to result in the identification of one additional Gram-positive bacterial specimen. NNT_GN_ was the number of PBCs containing Gram-negative bacteria that must be tested with one test versus another to result in the identification of one additional Gram-negative bacterial specimen. NNT_GP_ is calculated as the reciprocal of the difference in sensitivities for Gram-positive bacterial species identification, and NNT_GN_ is the reciprocal of the difference in sensitivities for Gram-negative bacterial species identification. NNT_GP_ and NNT_GN_ were calculated for each possible comparison.

#### Step 4: Plot weighted accuracy as a function of relative importance

Weighted accuracy is the overall accuracy adjusted for relative importance and prevalence and is calculated as follows:

Weighted accuracy = [*rp*(Gram-positive sensitivity) + (1−*p*)(Gram-negative sensitivity)] × (1/(*rp* +1−*p*)), where *r* = relative importance and *P* = prevalence of Gram-positive bacteria

Relative importance was the ratio between the importance of accurately identifying Gram-positive and Gram-negative bacteria. The value of relative importance can range from zero to one (0%–100%). Relative importance can be greater than one (100%) if the importance of accurately identifying Gram-positive bacteria is greater than Gram-negative bacteria.

#### Step 5: Display the difference in weighted accuracy as a function of relative importance and prevalence

Step 5 displays a graphic comparing the weighted accuracy of the three RDTs across varying relative importance and prevalence. The final graphic displays shaded regions where each RDT has the highest weighted accuracy with combinations of relative importance and prevalence.

### Determination of prevalence and relative importance

Pathogen prevalence and relative importance were estimated to determine the optimal RDT for UMMC using BED-FRAME. Pathogen prevalence was estimated as the proportion of Gram-positive and Gram-negative bacteria among non-duplicate PBC isolates using the 2021 antibiogram data from UMMC clinical microbiology laboratory. Relative importance was determined by a survey given to the UMMC antimicrobial stewardship committee consisting of infectious disease pharmacists, critical care physicians, hospitalists, infection preventionists, nurse practitioners, and clinical microbiology directors. Prior to distribution, the survey was reviewed and approved by the medical director of the clinical microbiology laboratory and infection control physicians. The survey was given to a total of 49 individuals and received 18 responses. The 18 respondents answered all the questions. To capture relative importance, the survey asked to “Assign points (out of a total 100) to each of the following attributes of accuracy of a diagnostic assay for bloodstream infections, based on their relative importance in your clinical practice.” The attributes included the accuracy of detecting six pathogen groups: *Staphylococcus aureus*, *Streptococcus* species, *Enterococcus* species, *Acinetobacter baumannii*, *P. aeruginosa*, and Enterobacterales. The relative importance ratio was calculated by dividing the subtotal of the three Gram-positive bacteria by the subtotal of the three Gram-negative bacteria.

### Survey to determine preferred diagnostic attributes

Using the survey given to the UMMC antimicrobial stewardship committee (Supplemental Information 2), preferred diagnostic attributes for RDTs for BSIs were also determined. Without revealing the names of RDTs, volunteers were asked to rank them (1–4) based on sensitivities of Gram-positive and Gram-negative bacteria and yeast, time-to-results, hands-on time, and whether the test determines resistance. The survey asked participants what characteristics of a diagnostic test were most important, the most important pathogens at UMMC, and acceptable time-to-results.

## RESULTS

### Study population

Of the 301 PBCs, 28 were polymicrobial and 273 were monomicrobial (105 Gram-positive bacteria, 157 Gram-negative bacteria, and 11 yeast).

### Diagnostic assay comparison using traditional methods

#### Sensitivity

For monomicrobial PBCs, the overall sensitivity for all three RDTs was 88% (241/273). However, sensitivities for species identification of Gram-positive bacteria, Gram-negative bacteria, and yeast for the three RDTs differed. FLAT MS showed sensitivities of 81% (85/105), 95% (149/157), and 64% (7/11) for Gram-positive bacteria, Gram-negative bacteria, and yeast, respectively. As previously shown, the sensitivities for Gram-positive bacteria, Gram-negative bacteria, and yeast were 78% (82/105), 96% (151/157), and 73% (8/11) for Sepsityper and 86% (90/105), 90% (141/157), and 91% (10/11) for BioFire BCID2, respectively. Sensitivities for individual species are summarized in Table S1.

For polymicrobial PBCs, FLAT MS completely identified 29% (8/28) and partially identified 71% (20/28) of polymicrobial PBCs. Sepsityper completely identified 11% (3/28) and partially identified 82% (23/28) of polymicrobial PBCs and BioFire. BCID2 completely identified 93% (26/28) and partially identified 7% (2/28) of polymicrobial PBCs.

#### Time-to-results

Time-to-results for each RDT were similar for Sepsityper, BioFire BCID2, and FLAT MS: 52 (±5.2) minutes, 65 (±1.0) minutes, and 60 (±2.8) minutes, respectively.

#### Hands-on time

Hands-on time was longer (40 minutes ±2.8 minutes) for Sepsityper compared to 10 minutes or less for BioFire BCID2 and FLAT MS.

### BED-FRAME

#### Step 1: Displaying expected yield as a function of prevalence

**Fig 2:**
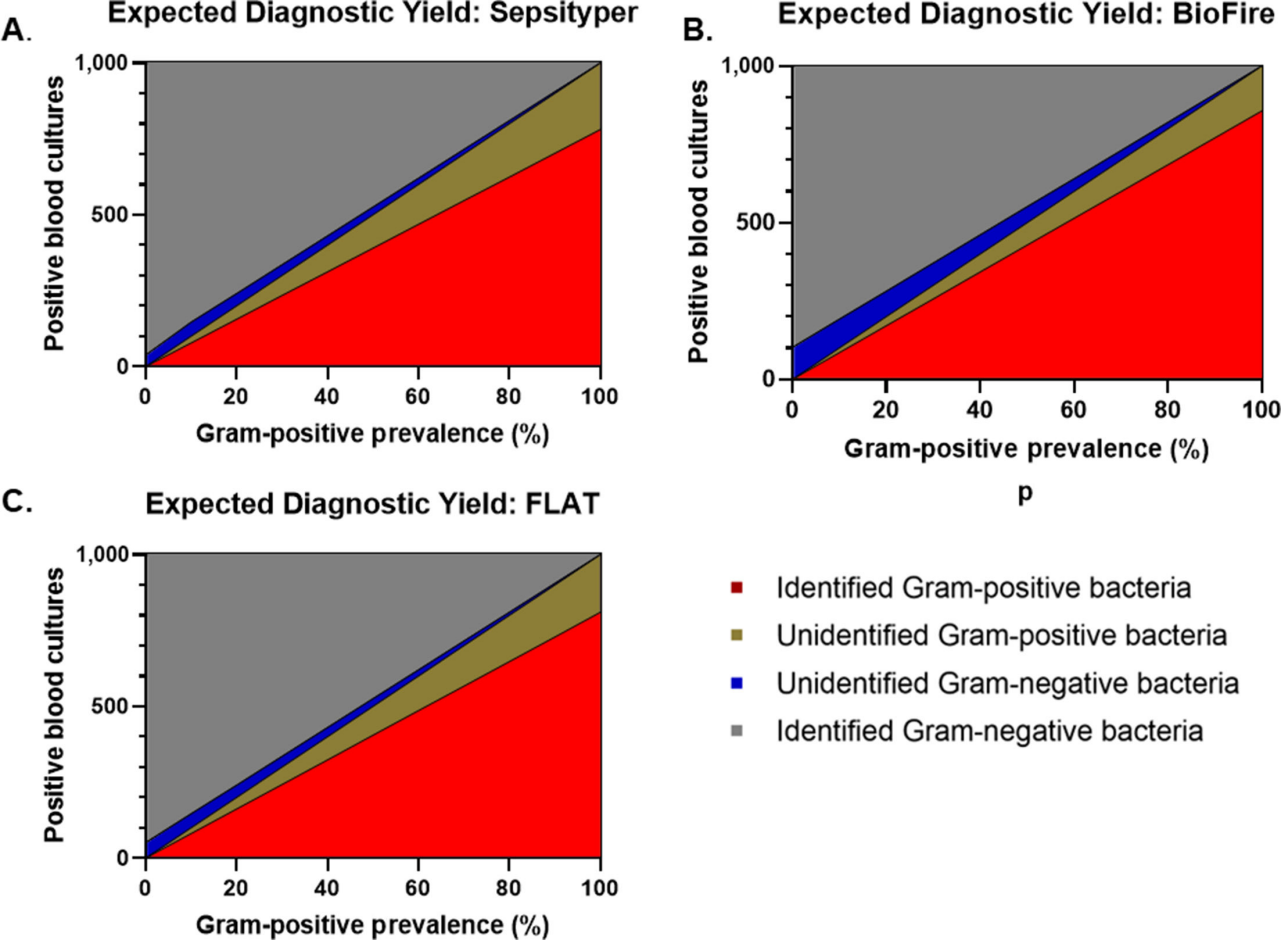
The expected diagnostic yield as a function of the prevalence of Gram-positive bacteria for a fixed population of 1,000 positive blood cultures for (**A**) Sepsityper, (**B**) BioFire BCID2, and (**C**) Fast Lipid Analysis Technique (FLAT MS). Expected diagnostic yield displays the distribution of accurately identified Gram-positive bacteria (red), accurately identified Gram-negative bacteria (gray), unidentified Gram-positive bacteria (gold), and unidentified Gram-negative bacteria (blue).

This step communicates the clinical impact of each RDT by displaying the expected diagnostic yield as a function of prevalence (Fig. 2A through C). When the prevalence of Gram-positive bacteria is 60%, the diagnostic yield for FLAT MS at a fixed population of *n* = 1,000 is as follows: accurately identified Gram-positive bacteria = 486, accurately identified Gram-negative bacteria = 379.6, unidentified Gram-positive bacteria = 114, and unidentified Gram-negative bacteria = 20.4.

#### Step 2: Plot the expected diagnostic difference in unidentified Gram-negative bacteria and accurately identify Gram-positive bacteria as a function of prevalence

**Fig 3:**
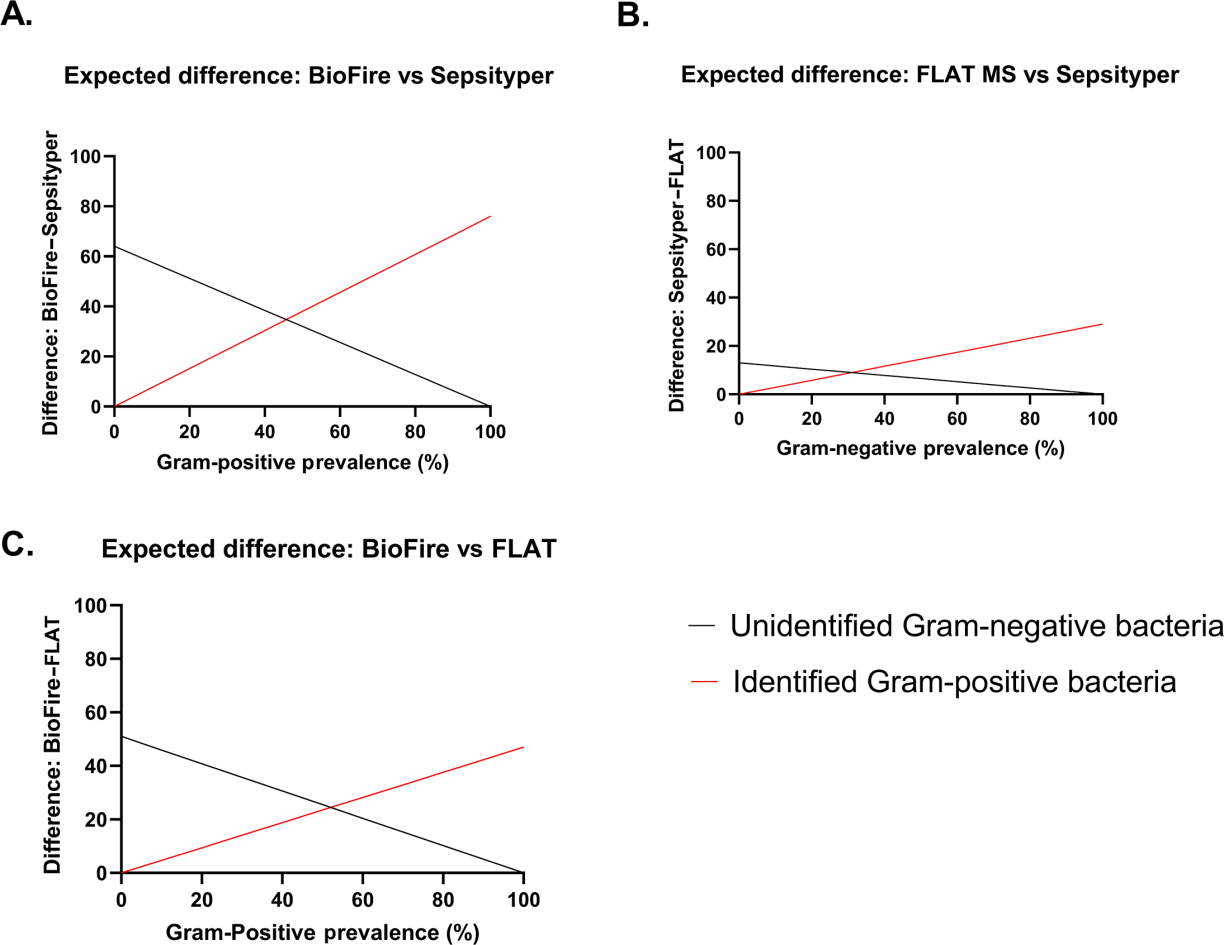
The between platform differences in unidentified Gram-negative bacteria (black) and accurately identified Gram-positive bacteria (red) raw numbers as a function of Gram-positive bacteria prevalence for all possible comparisons: (**A**) BioFire BCID2 vs Sepsityper, (**B**) FLAT MS vs Sepsityper, and (**C**) BioFire BCID2 vs Fast Lipid Analysis Technique (FLAT MS).

To highlight trade-offs between two RDTs, the expected diagnostic differences in raw numbers for unidentified Gram-negative bacteria and accurately identified Gram-positive bacteria were plotted as a function of Gram-positive prevalence for each combination of RDTs (Fig. 3A through C). The intersection points indicate the prevalence where trade-offs were equivalent. The Gram-positive prevalence for each intersection was approximately 45%, 36%, and 57% for BioFire versus Sepsityper, FLAT MS versus Sepsityper, and BioFire versus FLAT MS, respectively.

#### Step 3: Calculate NNT

NNT_GP_ was 13.2 (BioFire vs Sepsityper), 34.5 (FLAT MS vs Sepsityper), and 21.3 (BioFire vs FLAT MS); NNT_GN_ was 15.6 (Sepsityper vs BioFire), 76.9 (Sepsityper vs FLAT MS), and 19.6 (FLAT MS vs BioFire).

#### Step 4: Plot weighted accuracy as a function of relative importance

**Fig 4:**
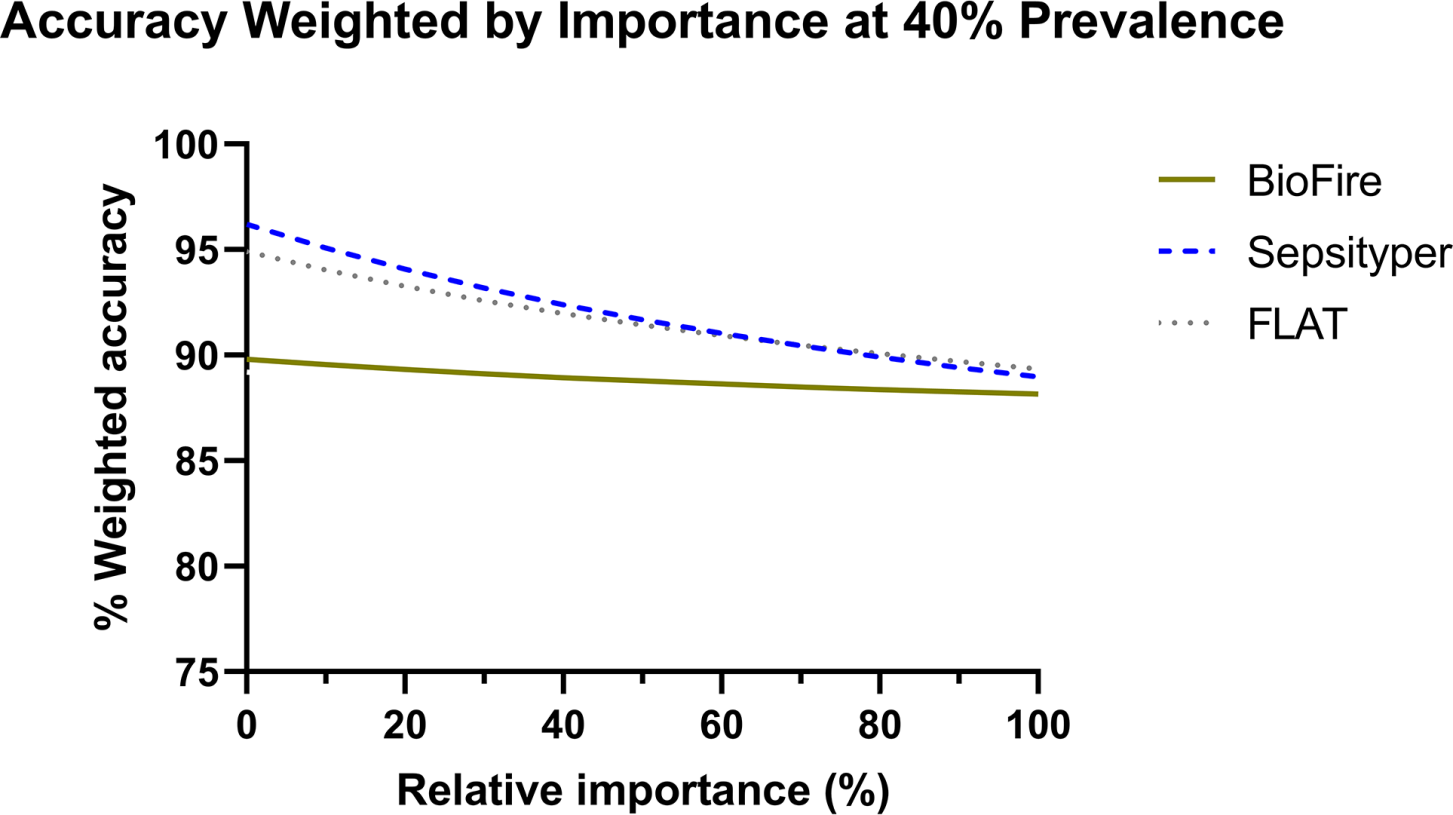
An example of weighted accuracy as a function of relative importance for each diagnostic test evaluated in this study. Weighted accuracies above were calculated at a prevalence of 40%. FLAT, Fast Lipid Analysis Technique.

Weighted accuracy of all three RDTs was plotted as a function of the relative importance of identifying Gram-positive versus Gram-negative bacteria. A theoretical example is shown when the prevalence of Gram-positive bacteria is 40% (Fig. 4). This step can be computed at any prevalence.

#### Step 5: Display the difference in weighted accuracy as a function of relative importance and prevalence

**Fig 5:**
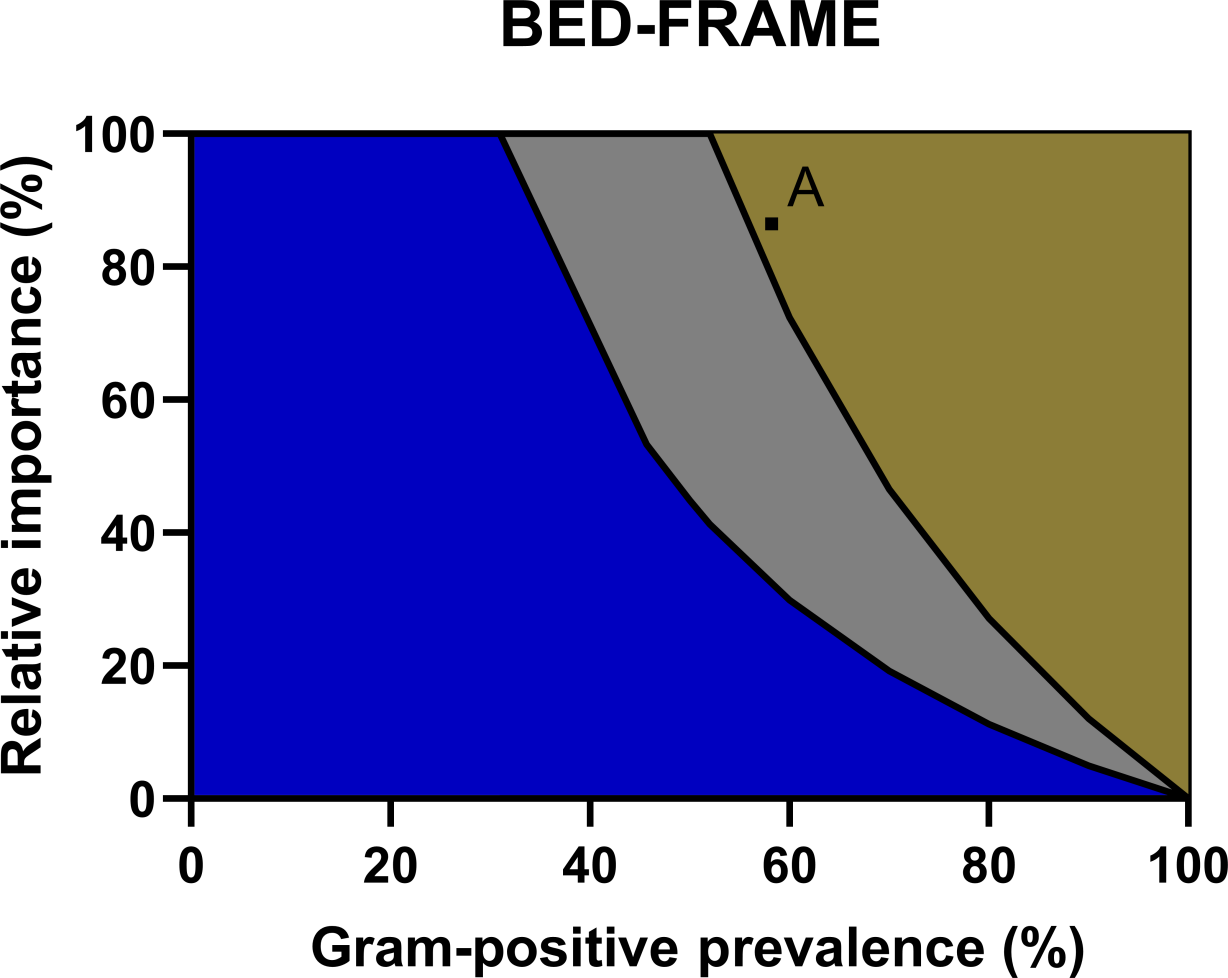
The figure displays a comparison of weighted accuracy as a function of the relative importance and prevalence of Gram-positive bacteria. Blue indicates combinations of relative importance and prevalence where Sepsityper is favored, gold where BioFire BCID2 is favored, and gray where FLAT MS is favored. The black curves represent where the two adjacent tests are equivalent. Point A represents the combination of relative importance and prevalence that represents UMMC.

Figure 5 provides a graphical comparison of weighted accuracy for all three RDTs as a function of relative importance and prevalence of Gram-positive bacteria. Blue indicates combinations of relative importance and prevalence where Sepsityper is favored, gold where BioFire is favored, and gray where FLAT MS is favored. The black curves represent where the two adjacent tests are equivalent.

### Determining optimal RDT using BED-FRAME

Among the assays compared in this study, the optimal RDT for UMMC was determined using the BED-FRAME results. Based on the 2021 antibiogram, the prevalence of Gram-positive bacteria among all non-duplicate PBCs was approximately 60%. Relative importance was determined using the results of the survey question that asked to score, out of a total of 100, the relative importance of identifying six groups of pathogens: *S. aureus*, *Streptococcus* species, *Enterococcus* species, *A. baumannii*, *P. aeruginosa*, and Enterobacterales. The average assigned values were 24.9, 7.2, 12.9, 15.6, 22.5, and 16.9, respectively. Subtotals for Gram-positive and Gram-negative bacteria were 45 and 55, respectively. Relative importance, the ratio of importance identifying Gram-positive to Gram-negative bacteria, was 0.815 (81.5%). Based on the prevalence of Gram-positive bacteria and the relative importance, BioFire was determined the best for UMMC followed by FLAT MS (Fig. 5).

### Preferred diagnostic attributes based on survey

BioFire BCID2 was ranked first by every respondent. FLAT MS had an average ranking of 2.2 (±0.41), Sepsityper had an average ranking of 3.2 (±0.71), and short-term culture, not a rapid test and therefore not included in this comparison, was last with an average ranking of 3.6 (±0.5). Participants preferred a more accurate but slower (83.3%) compared to a faster and less accurate (11.1%) test, and 5.6% had no preference. No respondents preferred a diagnostic test with time-to-results over four hours from PBC with 11.1% preferred results in approximately one hour or less, 27.8% within two hours, and 61.1% within four hours. All three RDTs compared in this study yielded average time-to-results in approximately one hour, meeting the preference of all respondents.

The most important diagnostic features were accurate pathogen identification (77.8%), identifying potential resistance (16.7%), and speed/time-to-results (5.6%). All three RDTs had similar overall accuracy and time-to-results. However, only BioFire BCID2 can identify resistance genes. Identifying polymicrobial PBCs and reducing hands-on time were not a priority. Most (94%) of the respondents indicated cost as the least important. When asked about the top three pathogens of concern at UMMC, the most common responses were *P. aeruginosa,* Enterobacterales, and *S. aureus*.

## DISCUSSION

This study compared our in-house method FLAT MS to two FDA-cleared, direct from PBC assays, Sepsityper and BioFire BCID2, using traditional evaluation methods and BED-FRAME for implementation at our academic medical center. Overall sensitivity for monomicrobial PBCs was equivalent for all three assays. Although accurate identification of Gram-positive and Gram-negative bacteria differed, each test had time-to-results of approximately one hour, a major benefit as shorter time-to-results potentially improves patient outcomes (14). Despite similar time-to-results, hands-on time differed. While FLAT MS and BioFire BCID2 had minimal hands-on time, Sepsityper had a hands-on time of approximately 40 minutes, which may prevent burdened or understaffed laboratories from utilizing Sepsityper (15). Despite similar hands-on time, the workflow of FLAT MS and BioFire BCID2 differ as BioFire BCID2 provides a more “walk-away” workflow. Additionally, although this study primarily focused on monomicrobial PBCs, it is important to note BioFire BCID2 outperformed the other platforms when identifying polymicrobial PBCs. Cost was not formally evaluated, but FLAT MS has shown to be a cost-effective method that is accurate and rapid, even though initial costs to implement FLAT MS including different reagents and diagnostic software, exist. However, these costs are common for any emerging technology in the clinical laboratories.

Despite thorough comparison using traditional methods, choosing which of these RDTs is best may remain difficult. Traditional calculations are limited as they treat different diagnostic attributes as equally important and are dependent on pathogen prevalence of the study. BED-FRAME aids in the decision-making by comparing the accuracy of these tests while considering pathogen prevalence and relative importance to the provider. The BED-FRAME analysis for this study demonstrated that each test has utility depending on pathogen prevalence and relative importance assigned by an institution. The estimated prevalence of PBCs containing Gram-positive bacteria, globally, is 50% to 75% (16–18). Given this, Sepsityper would only be the preferred test at low relative importance for Gram-positive bacteria (<25%) while BioFire BCID2 and FLAT MS would be optimal for any relative importance (>25%) depending on whether prevalence was on the lower or higher end of the published range. Moreover, at the time of this study, BioFire BCID2 was the mainstay technology for identification of microbes directly from PBC. BED-FRAME further confirmed the use of BioFire BCID2 in clinical laboratories.

BED-FRAME addresses the major limitations of comparing diagnostics; however, several weaknesses remain. For instance, BED-FRAME does not consider important components such as time and cost. Because of this, we recommend that BED-FRAME be conducted only when comparing diagnostics with similar time-to-results, as in this study. Additional information provided by the survey further assisted decision-making even when applying the results of BED-FRAME. While FLAT MS was second based on the results of BED-FRAME, consumables cost for FLAT MS analysis was significantly less than BioFire BCID2 (19–21). Thus, factors such as cost may lead certain healthcare settings to choose FLAT MS over BioFire BCID2 despite BioFire BCID2 being preferred according to BED-FRAME; however, cost was a low priority at UMMC. Furthermore, if FLAT MS was determined to be the preferred test followed by BioFire BCID2, the rankings from the survey and priority of resistance identification may lead to choosing BioFire BCID2.

Overall, this study serves as a proof-of-concept for comparing diagnostics using BED-FRAME and survey comparing the perceived value of additional test attributes. Prior to this study, this framework was proposed using a theoretical scenario and has yet to be applied in a published study. Moreover, this study provides a method for capturing relative importance through survey. The use of a survey in combination with BED-FRAME in this study provides a streamlined framework for other healthcare settings deciding between diagnostic tests.

## SUPPLEMENTAL MATERIAL

Supplemental S1 and S2 jcm.01096-23-s0001.docx Supplemental table 1 with organism identification rate and S2 with survey results.

ASM does not own the copyrights to Supplemental Material that may be linked to, or accessed through, an article. The authors have granted ASM a non-exclusive, world-wide license to publish the Supplemental Material files. Please contact the corresponding author directly for reuse.
