# Supplementary material for: Comparison of three rapid diagnostic tests for bloodstream infections using Benefit-risk Evaluation Framework (BED-FRAME)
Source: J Clin Microbiol. 2023 Dec 6;62(1):e01096-23. doi: 10.1128/jcm.01096-23 (PMC10793330; doi:10.1128/jcm.01096-23)
Supplement: Supplemental S1 and S2 — Supplemental table 1 with organism identification rate and S2 with survey results. [file jcm.01096-23-s0001.docx]

**Supplemental Table 1.**

| Species | N | Sepsityper | % | BioFire | % | FLAT MS | % |
| --- | --- | --- | --- | --- | --- | --- | --- |
|  |  |  |  |  |  |  |  |
| **Gram-positive bacteria** | | | | | | | |
| *Staphylococcus epidermidis* | 24 | 21 | 87.5 | 24 | 100.0 | 22 | 91.7 |
| Other coagulase-negative *Staphylococcus* | 12 | 12 | 100.0 | 12 | 100.0 | 9 | 75.0 |
| *Staphylococcus aureus* | 23 | 21 | 91.3 | 23 | 100.0 | 20 | 87.0 |
| *Enterococcus faecalis* | 6 | 5 | 83.3 | 6 | 100.0 | 4 | 66.7 |
| *Enterococcus faecium* | 4 | 3 | 75.0 | 4 | 100.0 | 3 | 75.0 |
| *Streptococcus* species | 21 | 13 | 61.9 | 21 | 100.0 | 17 | 81.0 |
| *Cutibacterium acnes* | 1 | 0 | 0.0 | 0 | 0.0 | 0 | 0.0 |
| *Micrococcus* species | 5 | 2 | 40.0 | 0 | 0.0 | 4 | 80.0 |
| *Pediococcus* species | 1 | 0 | 0.0 | 0 | 0.0 | 0 | 0.0 |
| *Rothia* species | 3 | 3 | 100.0 | 0 | 0.0 | 3 | 100.0 |
| *Corynebacterium* species | 3 | 1 | 33.3 | 0 | 0.0 | 2 | 66.7 |
| *Bacillus* species | 2 | 1 | 50.0 | 0 | 0.0 | 1 | 50.0 |
| **Gram-negative bacteria** | | | | | | | |
| *Escherichia coli* | 44 | 43 | 97.7 | 44 | 100.0 | 44 | 100.0 |
| *Klebsiella pneumoniae* | 35 | 35 | 100.0 | 35 | 100.0 | 34 | 97.1 |
| *Klebsiella oxytoca* | 6 | 6 | 100.0 | 6 | 100.0 | 6 | 100.0 |
| *Klebsiella aerogenes* | 4 | 4 | 100.0 | 4 | 100.0 | 4 | 100.0 |
| *Enterobacter cloacae* complex | 15 | 14 | 93.3 | 15 | 100.0 | 15 | 100.0 |
| *Proteus* species | 4 | 4 | 100.0 | 4 | 100.0 | 4 | 100.0 |
| *Pseudomonas aeruginosa* | 17 | 16 | 94.1 | 17 | 100.0 | 17 | 100.0 |
| *Pseudomonas putida* | 2 | 2 | 100.0 | 0 | 0.0 | 2 | 100.0 |
| *Moraxella* species | 1 | 0 | 0.0 | 0 | 0.0 | 1 | 100.0 |
| *Pantoea* species | 1 | 0 | 0.0 | 0 | 0.0 | 0 | 0.0 |
| *Citrobacter koseri* | 1 | 1 | 100.0 | 0 | 0.0 | 1 | 100.0 |
| *Citrobacter freundii* complex | 3 | 3 | 100.0 | 0 | 0.0 | 3 | 100.0 |
| *Providencia rettgeri* | 1 | 1 | 100.0 | 0 | 0.0 | 1 | 100.0 |
| *Bacteroides thetaiotaomicron* | 1 | 1 | 100.0 | 0 | 0.0 | 1 | 100.0 |
| *Bacteroides fragilis* | 2 | 2 | 100.0 | 2 | 100.0 | 2 | 100.0 |
| *Capnocytophaga* species | 1 | 1 | 100.0 | 0 | 0.0 | 1 | 100.0 |
| *Delftia* species | 1 | 1 | 100.0 | 0 | 0.0 | 0 | 0.0 |
| *Veillonella* species | 1 | 1 | 100.0 | 0 | 0.0 | 1 | 100.0 |
| *Haemophilus influenzae* | 2 | 1 | 50.0 | 2 | 100.0 | 1 | 50.0 |
| *Pasteurella multocida* | 1 | 1 | 100.0 | 0 | 0.0 | 0 | 0.0 |
| *Stenotrophomonas maltophilia* | 1 | 1 | 100.0 | 1 | 100.0 | 1 | 100.0 |
| *Leclercia* species | 1 | 1 | 100.0 | 0 | 0.0 | 0 | 0.0 |
| *Tissierella* species | 1 | 1 | 100.0 | 0 | 0.0 | 0 | 0.0 |
| *Serratia marcescens* | 11 | 11 | 100.0 | 11 | 100.0 | 10 | 90.9 |
| **Yeast** | | | | | | | |
| *Candida albicans* | 2 | 2 | 100.0 | 2 | 100.0 | 1 | 50.0 |
| *Candida glabrata* | 5 | 3 | 60.0 | 5 | 100.0 | 2 | 40.0 |
| *Candida parapsilosis* | 3 | 3 | 100.0 | 3 | 100.0 | 3 | 100.0 |
| *Candida lusitaniae* | 1 | 0 | 0.0 | 0 | 0.0 | 1 | 100.0 |
| ***Total*** | **273** | **241** | **88.3** | **241** | **88.3** | **241** | **88.3** |

**Supplemental information 2.**

Survey questions

1. Rank the following diagnostic tests based on their performance results.
   1. Test A
      1. Gram-positive bacteria sensitivity: 78.1%
      2. Gram-negative bacteria sensitivity: 96.2%
      3. Yeast sensitivity: 72.7%
      4. Time to results: 52 minutes
      5. Hands-on time: 40 minutes
      6. Resistance not detected
   2. Test B
      1. Gram-positive bacteria sensitivity: 83.8%
      2. Gram-negative bacteria sensitivity: 89.2%
      3. Yeast sensitivity: 0.0%
      4. Time to results: 5 hours
      5. Hands-on time: 4 minutes
      6. Resistance not detected
   3. Test C
      1. Gram-positive bacteria sensitivity: 81.0%
      2. Gram-negative bacteria sensitivity: 94.9%
      3. Yeast sensitivity: 63.4%
      4. Time to results: 60 minutes
      5. Hands-on time: 10 minutes
      6. Resistance not detected
   4. Test D
      1. Gram-positive bacteria sensitivity: 85.7%
      2. Gram-negative bacteria sensitivity: 89.8%
      3. Yeast sensitivity: 90.9%
      4. Time to results: 65 minutes
      5. Hands-on time: 5 minutes
      6. Resistance detected

| **Diagnostic test** | **Average ranking (+/-)** |
| --- | --- |
| BioFire® BCID2 (Test D) | 1.0 (+/- 0) |
| Short-term culture (Test B) | 3.6 (+/- 0.5) |
| FLAT MS (Test C) | 2.2 (+/- 0.41) |
| Sepsityper® (Test A) | 3.2 (+/- 0.71) |

1. Please assign points (out of a total 100) to each of the following attributes of accuracy of a diagnostic assay for bloodstream infections, based on their relative importance in your clinical practice. **The total should not exceed 100 points**.
   1. Accurate detection of *Staphylococcus aureus*
   2. Accurate detection of *Streptococcus* species
   3. Accurate detection of *Enterococcus* species
   4. Accurate detection of *Acinetobacter baumannii*
   5. Accurate detection of *Pseudomonas aeruginosa*
   6. Accurate detection of Enterobacterales

| **Microorganism** | **Average relative importance** | **Standard deviation** |
| --- | --- | --- |
| *S. aureus* | 24.9 | 8.3 |
| *Streptococcus* species | 7.2 | 6.2 |
| *Enterococcus* species | 12.9 | 6.5 |
| *A. baumannii* | 15.6 | 6.3 |
| *P. aeruginosa* | 22.5 | 6.1 |
| Enterobacterales | 16.9 | 4.6 |

Gram-positive bacteria subtotal: 44.9

Gram-negative bacteria subtotal: 55.1

1. Please assign points (out of a total 100) to each of the following attributes of resistance detection by a diagnostic assay for bloodstream infections, based on their relative importance in your clinical practice. **The total should not exceed 100 points.**
   1. Carbapenemases
   2. ESBL
   3. Colistin resistance
   4. Methicillin resistance
   5. Vancomycin resistance

| **Resistance** | **Average relative importance** | **Standard deviation** |
| --- | --- | --- |
| Carbapenemases | 25.4 | 9.6 |
| ESBL | 24.5 | 6.7 |
| Colistin resistance | 2.0 | 1.9 |
| Methicillin resistance | 29.5 | 12.7 |
| Vancomycin resistance | 18.7 | 7.9 |

1. Describe your preference when considering time and accuracy trade-offs
   1. I would rather have a faster test that is less accurate
   2. I would rather have a slower test that is more accurate
   3. I have no preference between time and accuracy


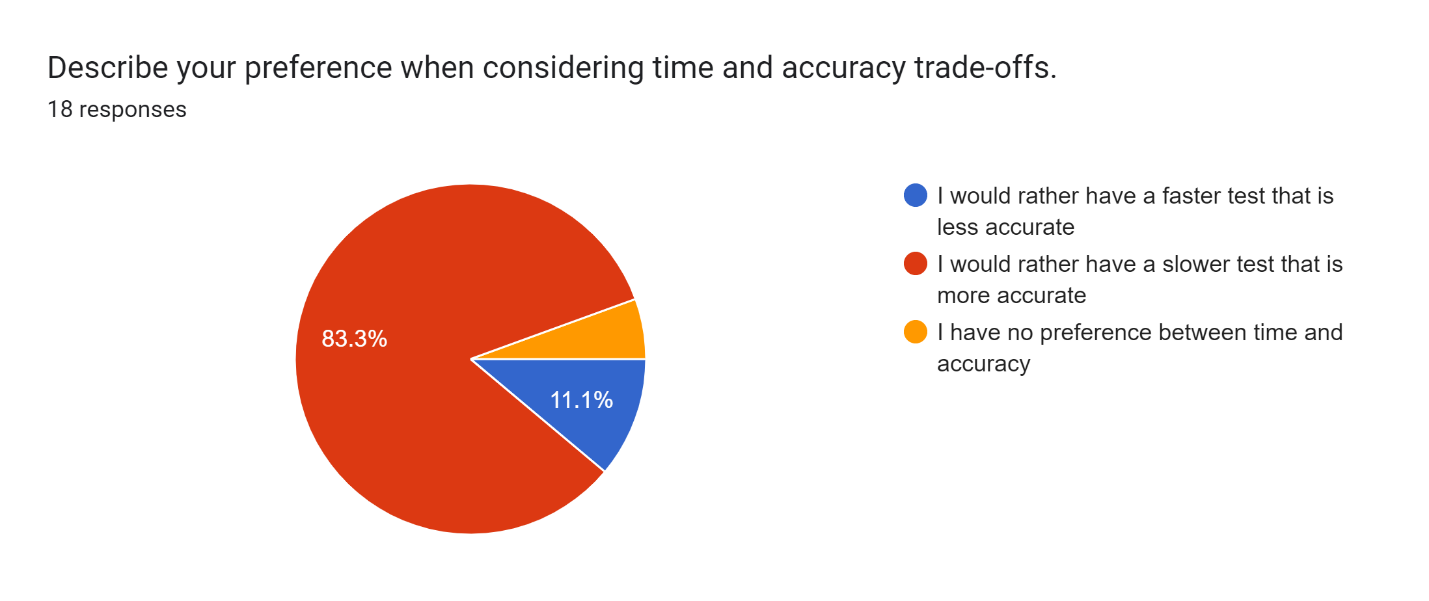


5.6%

1. What is an acceptable time to results for a rapid direct from blood culture diagnostic?
   1. Approximately one hour or less after positive blood culture
   2. Within 2 hours of positive blood culture
   3. Within 4 hours of positive blood culture
   4. Within 8 hours of positive blood culture
   5. Within 24 hours of positive blood culture


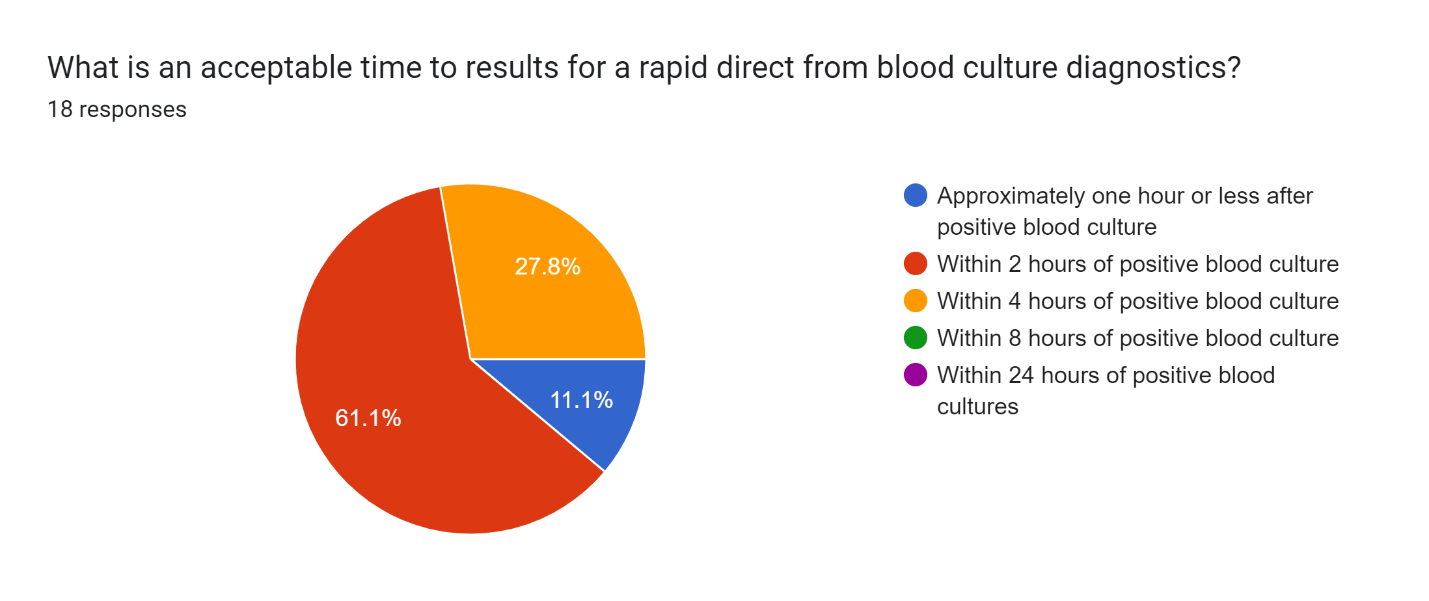


1. For blood culture diagnostics, which feature is most important?
   1. Identifying potential resistance
   2. Accurately identifying important pathogen species
   3. Speed and time to results
   4. Identification of polymicrobial infections
   5. Reduced labor and effort of laboratory staff


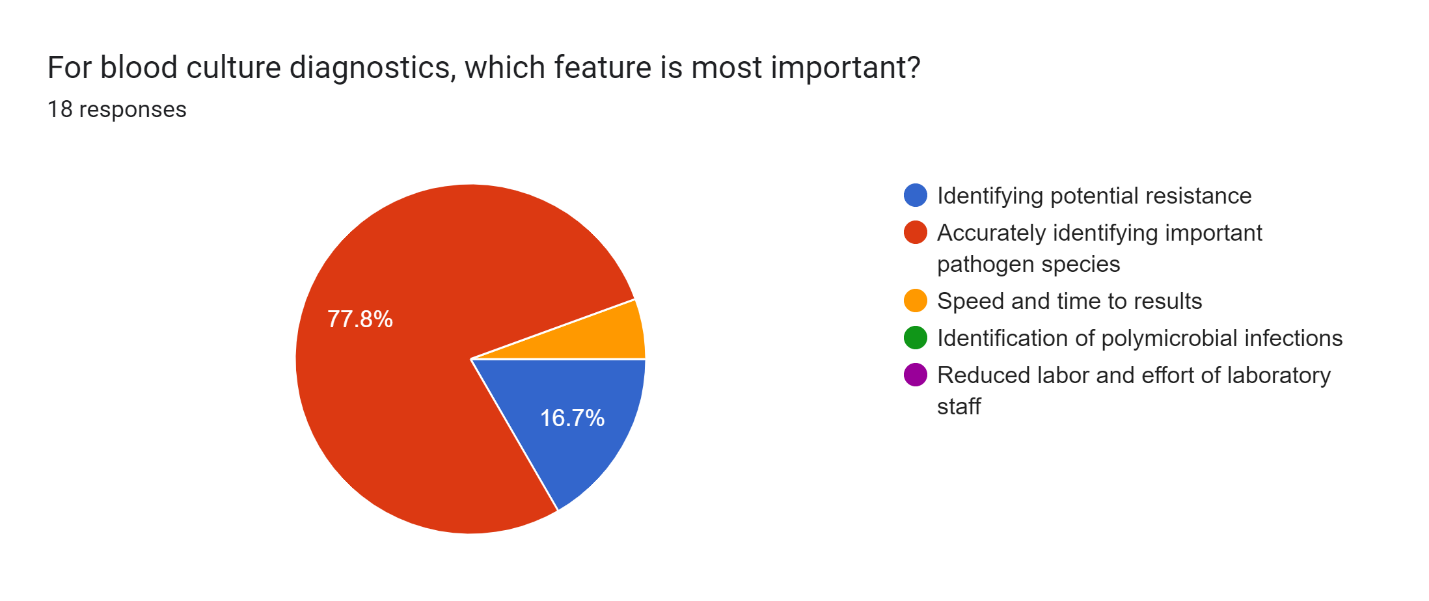


5.6%

1. In order of importance, which features of a diagnostic test are most important?
   1. Cost, time, accuracy
   2. Time, cost, accuracy
   3. Time, accuracy, cost
   4. Accuracy, time, cost
   5. Accuracy, cost, time


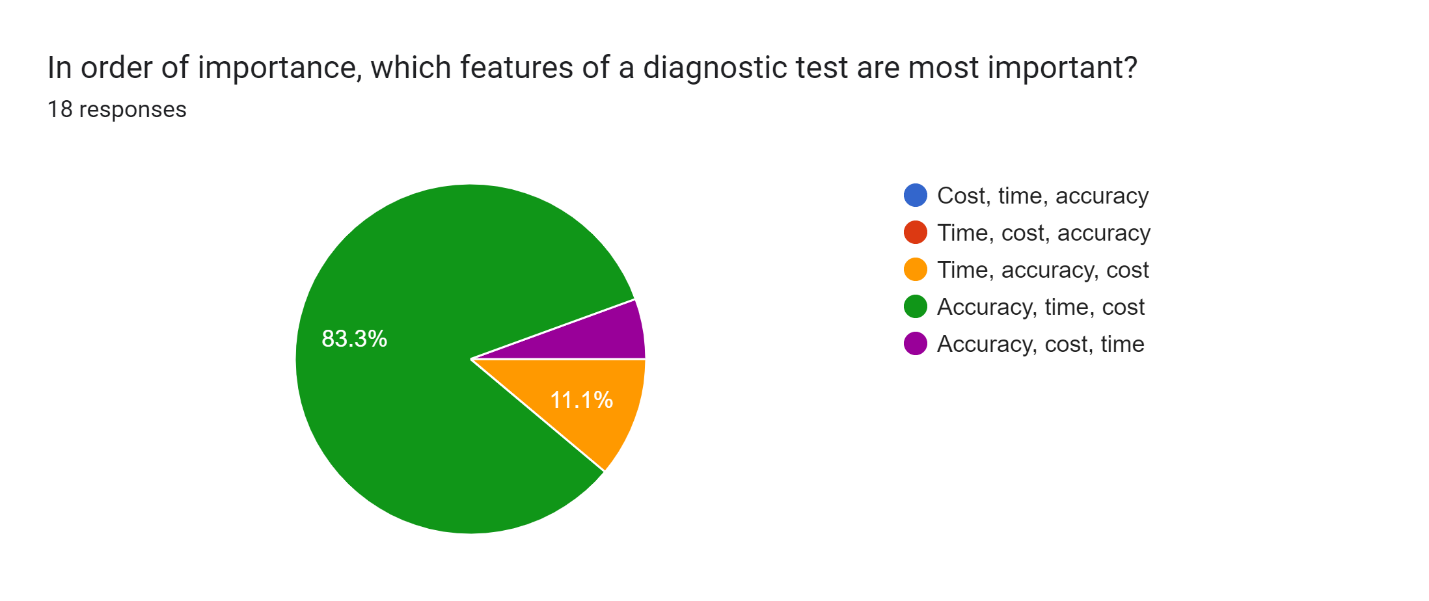


5.6%

1.
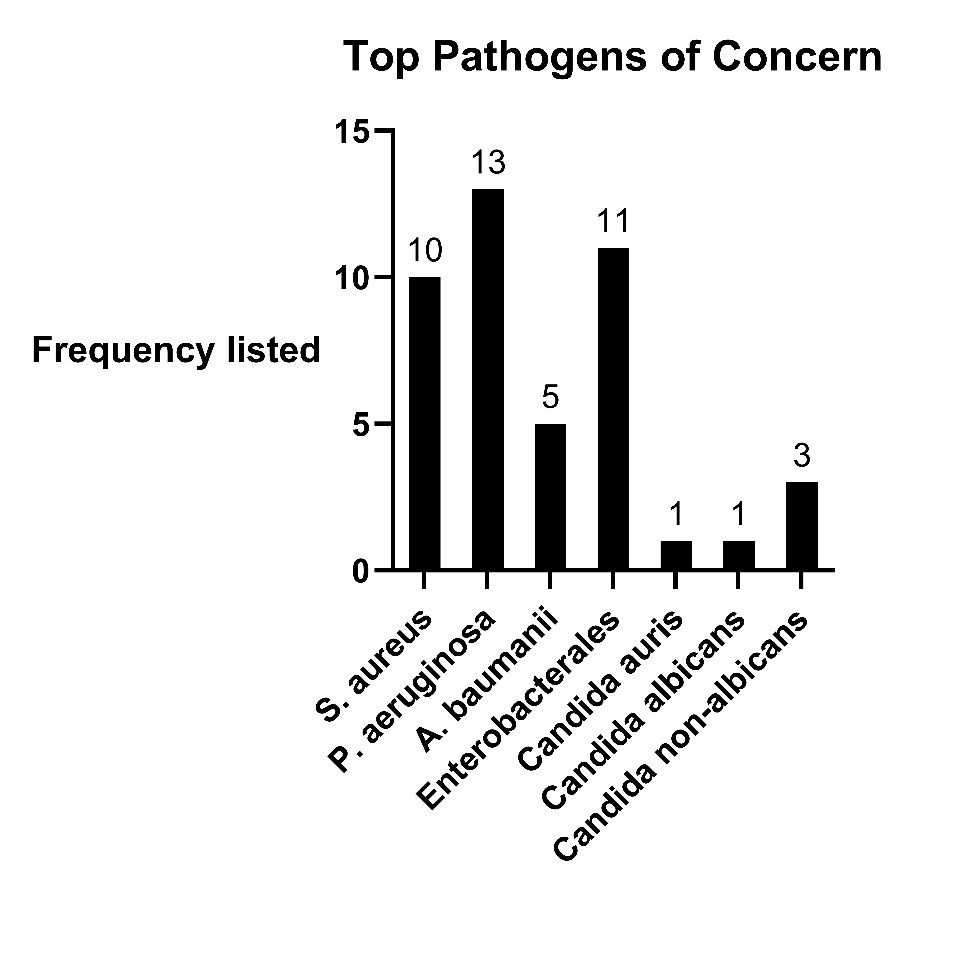
Please list the top 3 pathogens of concern at the UMMC hospital.
